# Intra-rater Kappa Accuracy of Prototype and ICD-10 Operational Criteria-Based Diagnoses for Mental Disorders: A Brief Report of a Cross-Sectional Study in an Outpatient Setting

**DOI:** 10.3389/fpsyt.2022.793743

**Published:** 2022-03-02

**Authors:** Helio G. Rocha Neto, Tomas Boldrini Sinem, Luisa Mendez Koiller, Amanda Machado Pereira, Bianca Marques de Souza Gomes, Carlos Linhares Veloso Filho, Maria T. Cavalcanti, Diogo Telles-Correia

**Affiliations:** ^1^Programa de Pós Graduação em Psiquiatria e Saúde Mental – PROPSAM, Instituto de Psiquiatria, Universidade Federal do Rio de Janeiro, Rio de Janeiro, Brazil; ^2^Programa de Doutoramento do Centro Acadêmico de Medicina da Universidade de Lisboa – CAMLPHD, Lisbon, Portugal; ^3^Medicine Faculty, Centro de Ciências da Saúde, Universidade Federal do Rio de Janeiro, Rio de Janeiro, Brazil; ^4^Instituto de Psiquiatria, Universidade Federal do Rio de Janeiro, Rio de Janeiro, Brazil; ^5^Clinica Universitária de Psiquiatria e Psicologia Médica, Faculdade de Medicina, Universidade de Lisboa, Lisbon, Portugal

**Keywords:** diagnosis, reliability and validity, ICD-10, mental disorders, prototype, categorical diagnosis, dimensional diagnosis, bias

## Abstract

**Background and Objectives:**

The use of “operational criteria” is a solution for low reliability, contrasting with a prototypical classification that is used in clinics. We aim to measure the reliability of prototypical and ICD-10 diagnoses.

**Methods:**

This is a retrospective study, with a convenience sample of subjects treated in a university clinic. Residents reviewed their diagnosis using ICD-10 criteria, and Cohen's kappa statistic was performed on operational and prototype diagnoses.

**Results:**

Three out of 30 residents participated, reviewing 146 subjects under their care. Diagnoses were grouped in eight classes: organic (diagnoses from F00 to F09), substance disorders (F10–F19), schizophrenia spectrum disorders (F20–F29), bipolar affective disorder (F30, F31, F34.0, F38.1), depression (F32, F33), anxiety-related disorders (F40–F49), personality disorders (F60–F69), and neurodevelopmental disorders (F70–F99). Overall, agreement was high [*K* = 0.77, 95% confidence interval (CI) = 0.69–0.85], with a lower agreement related to personality disorders (*K* = 0.58, 95% CI = 0.38–0.76) and higher with schizophrenia spectrum disorders (*K* = 0.91, 95% CI = 0.82–0.99).

**Discussion:**

Use of ICD-10 criteria did not significantly increase the number of diagnoses. It changed few diagnoses, implying that operational criteria were irrelevant to clinical opinion. This suggests that reliability among interviewers is more related to information gathering than diagnostic definitions. Also, it suggests an incorporation of diagnostic criteria according to training, which then became part of the clinician's prototypes. Residents should be trained in the use of diagnostic categories, but presence/absence checking is not needed to achieve operational compatible diagnoses.

## Introduction

Unreliability among clinicians has haunted psychiatry at least since the 1940s (1). These problems continued to increase until they peaked during the 1970s, after the joint United States and United Kingdom schizophrenia reliability studies, which demonstrated a low agreement among North Americans and British psychiatrists for schizophrenia (2). Unreliability was a threat to evidence-based practice in psychiatry; thus, the use of operational criteria for diagnosis and structured diagnostic interviews (SDIs) were proposed as a solution, in addition to other changes to clinical practice and research (3).

Nowadays, all research in psychiatry is based on an operational criteria diagnosis, obtained through the use of SDIs, such as “The Structured Clinical Interview for Diagnostic and Statistical Manual of Mental Disorders” (SCID) (4), although neither SDIs nor operational checklists are used in everyday clinical practice (5). There are virtually no studies about DSM criteria use in clinical practice (6, 7), and many researchers advocate a return to a prototype-based diagnostic description (8, 9). Some authors view this decision as a return to a problematic paradigm used in the pre-DSM-III era (10, 11), despite the fact that well-built prototypes are both reliable (12) and clinically useful (13).

Typification and prototype elaboration are a natural way of reasoning. This process occurs through hypothesis testing, aggregating technical knowledge, and experience, representing the standard operation for a clinician to formulate a diagnosis (14). Thus, prototype-based diagnoses are probably what clinicians do in real-world mental healthcare scenarios, without the use of operational criteria and SDIs (15).

Although this may be true, prototype-based diagnoses are not necessarily the opposite of operationally based ones: prototypes are not only summing criteria until a positive diagnostic threshold is reached (16). Prototypes are mental models, built by clinicians based on their theoretical knowledge (e.g., ICD-10 operational criteria), clinical experience (e.g., observed patients), and observed patterns in a specific “kind” of subject (9, 15). Furthermore, these models are used as a standard for comparing new patients, during a subjective “fitness” evaluation. As an example, the comparison can be classified from 1 (not an example of that prototype) to 5 (the perfect example of that prototype), resulting in a prototype-based diagnosis (13).

We aimed to measure the intra-rater agreement of clinician prototype and ICD-10 operational criteria-based diagnosis, obtained through non-standardized diagnostic interviews (NSDI), in a real-life outpatient scenario. We hypothesize that a small disagreement will be detected and that the direct use and checks for the presence of ICD operational criteria might increase the number of identified diagnoses, due to the “checklist effect” (anti-anchoring bias strategy, which might bring more diagnostics to the fore) (17, 18). To check these hypotheses, we conducted a cross-sectional study at the outpatient clinics of the Psychiatric Institute of the Federal University of Rio de Janeiro (IPUB-UFRJ).

## Methods

We performed our study with a convenience sample, in a naturalistic, real-life outpatient setting, in an academic mental healthcare setting. The IPUB outpatient clinic is part of the mental healthcare apparatus of the UFRJ, providing treatment for a large catchment area in Rio de Janeiro, and more than 650 consultations monthly. Although it is considered a tertiary mental health facility in the Brazilian National Health Service (SUS), the patients' clinical conditions are very heterogeneous (19). The patient profile comprises chronic, recently admitted subjects or previously admitted patients under follow-up.

This is also the setting for the outpatient training portion of the psychiatry residency at UFRJ, where trainee psychiatrists practice diagnostic interviews and follow-up on patient attendance. In Brazil, the psychiatry residency is a 60-h week program and lasts for 3 years (20). At IPUB, residents provide outpatient assistance throughout their training, which means patients are usually followed by the same doctor for some years and, eventually, gain a new doctor when the former resident finishes the training period. Residents are the main work force in IPUB's outpatient clinics, and senior physicians do not have patients under their direct care; thus, the residents were the available option for prototype vs. operational intra-rater evaluation.

As a training scenario, every new doctor performs an entirely new diagnostic interview with each patient at the start of their tenure, but in an NSDI and not an SDI format. During the first semester of the course, residents learn about the use of ICD and DSM operational criteria for diagnosis, but criterion checking is not part of regular care. Instead of a diagnostic conclusion based on an operational checklist after a history-taking interview, residents usually create a non-standardized prototype for each disease, individually, based on a classification system and clinical lectures, assistance from supervising physicians, and inpatient and outpatient follow-up. As a real-life scenario, residents use these prototypes as a model to classify the patients and not operational criteria. This is an ideal setting to test the agreement of prototype and operational-based diagnosis, the first to be considered the index test, and the latter as a gold standard test.

Patients had already been prototype diagnosed by their clinicians, as a consequence of previous interviews and follow-up evaluations. Thus, residents were recruited and asked to review their clinical diagnosis by checking ICD-10 operational criteria.

Diagnostic training and experience in practice occur during the first year of residence. Consequently, clinicians included in this study had to be at least in the second year of the residence, and the prototype diagnoses based on more than 1 year of patient observation. Those two criteria were needed to guarantee a minimum clinical experience, disorder prototype development, and clinical and diagnostic class attendance. All 30 residents of the second and third years were invited to participate in the study. After acceptance, the subjects reviewed each of their patients' working diagnoses according to ICD-10 criteria (described below). The ICD-10 diagnostic system was chosen because it is the official diagnostic system for judicial and official reports in Brazil. It is also how diagnoses are registered in IPUB's patient profiles, and prototype-based diagnoses, although not operationally checked, are reported using the ICD-10 codes.

International Classification of Disease, Tenth Revision, operational-based diagnoses were achieved following these rules: The prototype-based diagnosis was the working diagnosis registered in the last follow-up consultation before operational criteria checking. After the regular follow-up consultation, the resident reviewed the patient file, considered the last interview, and then checked ICD-10 operational criteria for that disorder. To achieve a positive ICD-10 diagnosis, the minimum operational criteria must have been fulfilled (both qualitatively as a sign and symptom description and quantitatively with the number of criteria present), and all exclusion criteria applied, looking for differential diagnosis.

Neurodevelopmental and neurodegenerative diagnoses are usually persistent and directly affect clinical presentation for adult mental disorders (21). Thus, subjects with a diagnosis first observed during childhood (such as autism and mental impairment) or secondary to brain damage were considered to have these diagnoses independent of further developments. A subject suffering from psychosis who meets operational criteria for persistent delusional disorder, but that also has operational criteria for mental impairment, would then be classified as mentally impaired. Consequently, only one diagnosis was considered before and after the ICD-10 criterion application.

For statistical tests, the fifth ICD-10 digit descriptor was excluded, and only the main diagnosis was used to compare the number of diagnoses obtained before and after the ICD-10 operational criteria application. As the number of single diagnostic hypotheses would be too large for usual kappa measurement, diagnoses were grouped into the following subgroups: organic (diagnoses from F00 to F09), substance disorders (SD: F10–F19), schizophrenia spectrum disorders (SSD: F20–F29), bipolar affective disorder (BAD: F30, F31, F34.0, F38.1), depressive disorders (DD: F32, F33), anxiety-related disorders (ARD:F40–F49), personality disorders (PD: F60–F69), and neurodevelopmental disorders (ND: F70–F99).

Prototype and operational-based diagnoses were tabulated, listing diagnoses identified before and after ICD-10 operational criteria application. Where it was impossible to establish a single-prototype or ICD operational criteria-based diagnosis, the subject was excluded from the sample. All results were pooled and then used for statistics. Kappa intra-rater reliability tests were performed for each diagnosis, assuming other diagnoses as negative for the index test, and then reported as kappa index (accuracy), sensitivity, specificity, positive predictive value (PPV), and negative predictive value (NPV). As an example, when testing kappa for depression, ICD-10 operational-based diagnoses of schizophrenia were considered a true negative result for depression; therefore, only diagnoses of depression were considered as a true positive. Missing results were excluded from statistical analyses and reported as N/A wherever relevant.

EpiR library of R statistical software (22) was used to achieve a total kappa using all subgroups, and GraphPad online software (23) to measure kappa for each diagnosis. As a convenience sample study, the number of prototype diagnoses in that setting was unknown, and we could not find similar studies on which to base expected kappa; we did not calculate the sample size in advance. Assuming an agreement by chance with eight possible diagnoses, expected intra-rater agreement kappa before operational criteria application of 0.2 (no or low agreement), and a desired kappa of 0.8, we conducted an *ad hoc* calculation, reported in **Table 4**. Descriptive measurements were conducted in demographics characteristics using the R Commander package, with R software (24).

This study was assessed and approved by IPUB's ethics committee, as part of a larger diagnostic reliability study under development, registered under Certificate of Submission for Ethical Appraisal 33603220.1.0000.5263 and Universal Trial Number U1111-1260-1212, registered and approved by the Brazilian Clinical Trials Registry platform. All residents were invited to sign an informed consent, following ethical requirements for studies with human subjects.

## Results

Three out of 30 invited residents agreed to participate in the study, revising diagnoses from 146 patients. Two of the 146 tested subjects did not meet any ICD-10 operational criterion diagnosis and were excluded from the kappa analysis. All participant residents were female and in the second and third years of resident training. The diagnosis was obtained from patients of both genders (*n* = 146, 55.5% female), aged from 19 to 82 years [mean of 46.2 years, standard deviation (SD) = 16.1]. Most subjects were single and unemployed, with mean years of education of 9.89 years (SD = 3.05) (Table 1).

**Table 1:** Demographic characteristics.

**Minimum**	**Maximum**	**Mean**	**SD**	**Missing**
**Age (years)**				
19	82	46.2	16.1	44
**School years**				
0	13	9.89	3.05	40
**Gender**				
Female	Male	Other		
81 (55.5%)	64 (43.8%)	1 (0.68%)		
**Employment**				
Employed	Unemployed	Retired	Missing	
42	97	6	1	
**Marital status**				
Single	Married	Widowed	Divorced	
88	33	10	14	

*1: SD, standard deviation; N/A, not available*.

The number of diagnoses observed before and after the ICD-10 operational criteria application was registered and tested for statistical differences. We found 45 prototype-based diagnoses and 51 ICD-10 operational-based diagnoses using the entire four-digit ICD-10 descriptors. The independence Kruskal–Wallis *X*^2^-tests were used to compare the number of diagnoses before and after ICD-10 criteria application, and no statistically significant difference was found using either four or three digits (Table 2). After diagnosis conversion, pre- and post-diagnoses were grouped into eight subgroups, and we used a double-entry table for descriptive purposes (Table 3).

**Table 2:** Number of prototype vs. ICD-10 operational-based diagnosis.

**Diagnostic type**	**No. of** **diagnosis**	***X*** **^2^**	**df**	***p***
All diagnoses	Prototype	45	52.81	63	0.82
	ICD-10	51			
Main diagnoses	Prototype	20	21.75	26	0.70
	ICD-10	26			

**Table 3:** Prototype vs. ICD-10 operational-based diagnosis.

**ICD**	**ARD**	**BAD**	**DD**	**SSD**	**ND**	**Organic**	**PD**	**SD**	**Total**
**Prototype**									
ARD	16	1	1	0	2	0	2	0	22
BAD	1	28	1	1	0	0	2	0	33
DD	0	3	12	0	0	0	2	0	17
SSD	0	1	0	32	2	0	1	0	36
ND	0	0	0	0	14	0	0	0	14
Organic	0	0	0	0	0	2	0	0	2
PD	0	4	2	0	0	0	12	1	19
SD	0	0	0	0	0	0	0	1	1
Total	17	27	16	33	18	2	19	2	144

*ARD, anxiety-related disorders; BAD, bipolar affect disorder; DD, depressive disorder; SSD, schizophrenia spectrum disorder; ND, neurodevelopmental disorder; organic, brain damage or degenerative-related disorders; PD, personality disorders; SD, substance-related disorders*.

Intra-rater kappa measurements were at least moderate (kappa = 0.58) according to Cohen's kappa (25) and weak under McHugh's interpretation (26) in single disorders, although the overall kappa was high [overall kappa = 0.77, confidence interval (CI) 0.69–0.85]. As there were no healthy subjects in this sample, we could not calculate sensitivity, specificity, PPV, and NPV for the overall sample, but these values were high among individual diagnoses (Table 4).

**Table 4:** Agreement report of prototype vs. ICD-10 operational-based diagnosis, by diagnostic code.

**Diagnostic** **code**	**Kappa** **(SD)**	**Kappa** **(CI)**	**Sensitivity** **(CI)**	**Specificity** **(CI)**	**Positive predictive** **value (CI)**	**Negative predictive** **value (CI)**	**Minimum n for** **power >0.80**
Total	0.77	(0.69–0.85)	N/A	N/A	N/A	N/A	18
Organic	1	0	100%	100%	100%	100%	N/A
SSD	0.91 (0.82–0.99)	0.04	97%	96%	89%	99%	10
BAD	0.74 (0.61–0.84)	0.06	76%	95%	85%	92%	20
DD	0.69 (0.50–0.88)	0.1	75%	96%	71%	97%	25
ARD	0.79 (0.65–0.94)	0.08	94%	95%	73%	99%	17
PD	0.58 (0.38–0.76)	0.10	63%	94%	63%	94%	40
ND	0.86 (0.72–0.99)	0.07	78%	100%	100%	97%	12
SD	0.66 (0.05–1)	N/A	50%	100%	100%	99%	N/A

*ARD, anxiety-related disorders; BAD, bipolar affect disorder; DD, depressive disorder; SSD, schizophrenia spectrum disorder; ND, neurodevelopmental disorder; organic, brain damage or degenerative-related disorders; PD, personality disorders; SD, substance-related disorders; CI, confidence interval*.

## Discussion

The number of participating residents was small (10% of invited subjects), but similar to other studies in which recruitment was restricted to an electronic invitation (27). With three participants, any assumption about resident representativity and study generalizability is difficult; however, participating residents were of the same age and had the same clinical experience as the average resident in Brazil (28).

The diagnostic distribution in our sample was equivalent to previous studies in IPUB outpatient clinics (19) and can be considered representative of patients assisted in this setting. Initially, we assumed that residents would work with a small number of prototype-based diagnoses. However, we observed a spontaneous use of more than 40 diagnoses, inside almost all 10 ICD-10 mental health subgroups, except for the major group of behavioral syndromes associated with psychological disturbances and physical factors (F50–F59), which were not recorded at all. Clinically, all these diagnostics could be summarized in eight subcategories, reinforcing the hypothesis that psychiatric practice could be possible with fewer diagnostic constructs than presented in both manuals (29, 30).

As predicted, there was an increase in the number of final diagnoses after using ICD-10 operational criteria, although it was not statistically significant. Working and identifying only the most frequent diagnoses is a known bias in medicine (31), and the use of diagnostic checklists has been previously reported as an antibias solution (17, 18). Nevertheless, the final subgroups were the same among pre- and post-ICD-10 criteria application, so this increase might not be clinically relevant.

We expected the differences among pre- and post-ICD-10 criteria to be small, but the high intra-rater kappa size was impressive. Even the lower observed kappa (0.58) would be traditionally considered as a moderate agreement by Cohen (25). Consequently, we disagree with the use of operational criteria as a checklist during the diagnostic process since prototype elaboration usually achieves the same result. Based on these results, teaching the operational criteria to clinicians is enough for their inclusion in diagnostic prototypes.

The few subjects with organic and substance disorder diagnoses in this sample compromises analysis; however, sensitivity, specificity, PPV, and NPV for the other diagnoses were high. Except for PD, all other diagnostic groups had a PPV >70%, confirming prototype diagnoses for most cases. Also, NPV results indicate that residents using prototype-based diagnoses could identify when the disease was not present with more than 94% accuracy. In our study, a prototype diagnosis was usually right about the presence of operational diagnostic criteria for specific diagnostic groups, and almost always when not.

**Figure 1:**
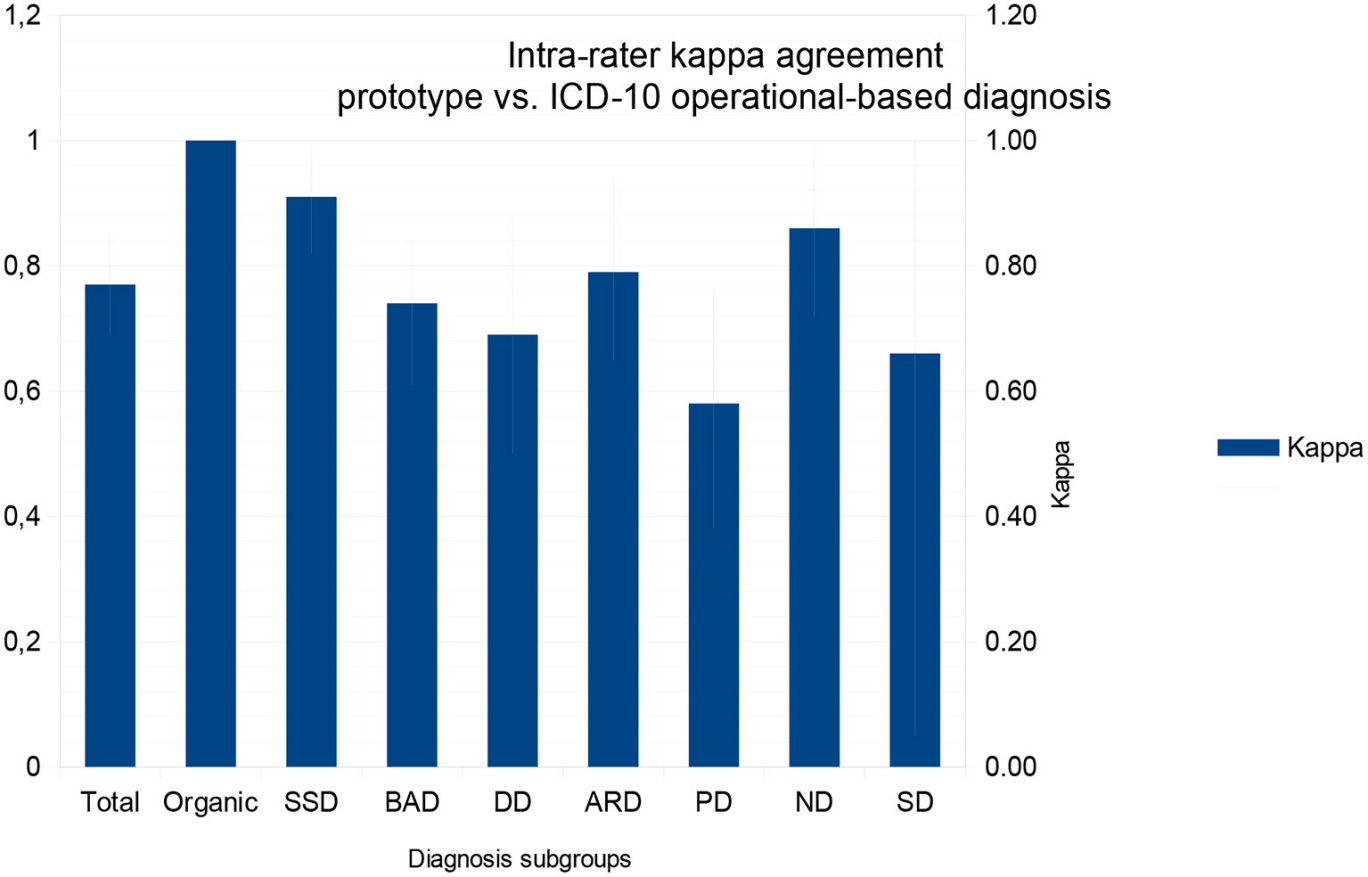
Kappa values between operational and prototype diagnostics for each diagnostic subgroup and for the total comparisons. Values range from 0 (random agreement) to 1 (perfect agreement), and standard deviation is represented in light blue. ARD, Anxiety Related Disorders; BAD, Bipolar Affect Disorder; DD, Depressive Disorder; SSD, Schizophrenia Spectrum Disorder; ND, Neurodevelopmental Disorder; Organic, Brain damage or degenerative related disorders; PD, Personality Disorders; SD, Substance related Disorder.

Diagnostic constructs in psychiatry have different levels of validity and agreement (32, 33), and our results support that assumption. Although all kappa values were high, DD and PD are harder to identify as a prototype than SSD and ND (Figure 1). These findings probably reflect that the SSD and ND criteria are strongly bound and easier to include in a prototype (34) than DD and PD.

During the reliability crisis, Helzer (3) hypothesized that an operational criteria definition was fundamental to increasing reliability among clinicians. However, we demonstrated that they do not need to be used as a checklist while in practice, since they have little effect on the clinician's opinion. Although this may be true, it is not the same to say that diagnostic operational criteria are irrelevant, since they may have an important effect on inter-rater agreement, not addressed in the present paper. A prototype is a broader diagnostic construct and may include operational criteria, even if not dependent upon them (35). Consequently, clinicians need to have operational descriptors for mental diagnoses to develop their prototypes for clinical practice. With well-built prototypes, they can reach the same conclusions as the operational system, without the need to check the presence or absence of descriptors in every diagnostic interview.

We were unable to check the effect of “sinking cost,” “anchorage,” and “self-satisficing” bias. These biases are operator-related and could only be avoided using an SDI or another interviewer. This strategy could have resulted in other diagnoses after diagnostic review (36). However, asking for another clinician to provide ICD-10 criterion diagnosis, through a new NSDI or SDI, would result in a different data-gathering strategy, adding another bias caused by the use of different “instruments” to access clinical data. What was measured here was the ability for a clinician to identify the described disorder in ICD-10 with a prototype-based diagnosis, but not if their strategy to obtain diagnostic information was precise. These biases are related to information gathering; a systematic approach in history-taking and mental status evaluation could provide diagnostic information relevant for a diagnostic change for both operational and prototype-based criteria.

The present study has four main limitations. First, as a way to avoid inter-rater bias, both the prototype and ICD-10 operational diagnose rater were the same. On the one hand, it avoids the problem of diagnosis based on different history-taking strategies and examination (information bias), but on the other, clinicians may resist changing their diagnosis, representing a “sinking cost” bias (when someone refuses to change a belief in face of new evidence). Besides that, our results fulfilled their objective to measure the effect of using diagnostic operational criteria in a checklist format for diagnostic intra-rater agreement and reproduce what would happen in a real clinical scenario. In other words, after a diagnostic NSDI, a clinician would reach a prototype diagnosis spontaneously before application of ICD-10 operational criteria (15) and may resist accepting an operational diagnose that does not match their earlier opinion. Consequently, sinking cost bias is unavoidable whether criteria application was used by the patients' clinician or not.

Second, the IPUB outpatient setting may not represent other care scenarios, even in Brazil, since it has an academic vocation that may result in differential clinical training. A high intra-rater kappa agreement could be the result of the unconscious incorporation of ICD-10 operational criteria, after repetitive classes in resident prototypes. Moreover, setting bias is related to the diagnostic sample since IPUB's research outpatient group takes most subjects with certain specific diagnoses during the admission triage. This results in a low prevalence of post-traumatic stress disorder, obsessive-compulsive disorder, social and specific phobic disorders, substance misuse disorder, dementia, and other advanced age-related disorders. Subjects identified with such a diagnosis during the admission process may be selected for research and are not regularly assisted by residents, so prevalence is not representative of other outpatient clinics. Also, IPUB outpatient clinics are fully manned by psychiatry residents, so we could not evaluate how senior physicians would behave in such prototype compared with operational diagnostic tests.

Third, we decided to not consider the possibility of clinical comorbidity and to adopt a hierarchical diagnostic structure with ICD-10 that does not reflect research practice, which usually accepts multiple comorbidities. We did so since neurodevelopment and organic and pediatric psychiatric diagnoses have an impact when evaluating adult disorders (21). Also, substance disorders are characterized by many behavioral changes that might be classified as PD, DD, ARD, or even BAD/SSD, but which are consequences of continued use of substances. It would be impossible to define, in the case of comorbidity, if these disorders were present before, during, or after the substance abuse. Also, the use of kappa instead of weighted kappa may make diagnosis disagreement seem worse than it would be in a clinical scenario (e.g., misdiagnosing DD as ARD has few consequences for medication decisions and the indication of psychotherapy).

Fourth, the number of participating residents raises questions about the generalization of our results. To address these limitations, it is necessary to explain the reasons why our study had only small participation of residents, and then discuss its consequences for kappa intra-rater statistics. First, the psychiatry residency is a 60-h/week course, IPUB residents have a personal caseload that varies from 80 to 120 in the outpatient clinics, and Brazilian law forbids additional payment for participation in research. That said, it is understandable that reviewing each outpatient diagnosis after consultation increases the workload, and only a few residents would be inclined to participate. Second, our study started to collect data during the COVID-19 pandemic, resulting in a non-standard functioning of outpatient clinics, so residents were less present in that setting. Third, due to an agreement with the ethics committee and COVID-related adaptations, all residents were invited only once, by email, which usually results in low participation.

Sample size in kappa studies is complex and requires three components to be calculated (expected natural agreement, expected to achieve agreement, and the number of possible variables/disorders); none of them related to the number of raters (37). Cohen described kappa when comparing two independent evaluators' opinions, or independent measurements by the same evaluator, without assumptions of the number of evaluators necessary for a valid affirmative or kappa use (25). Even in a modern and well-funded scenario, such as DSM-5 clinical trials, the greatest problem was the minimum number of subjects to be interviewed, and not the number of clinicians participating. Eight has been considered the minimum number of raters in DSM-5 clinical trials (38), probably because of the number of evaluations required and not inter-rater reliability concerns. Finally, generalizations are always difficult when a study is conducted at a single site, as local clinical routines and assisted patients would always be unique. Our results should be taken as a picture of that scenario, adding some evidence to the discussion about operational criteria checking effects in an already obtained prototype diagnosis. These results need to be replicated in other clinical scenarios to achieve appropriate validity.

## Conclusions

In our study, we have consistently shown prototype diagnosis as being reliable compared with a checklist operational system. Prototypes were demonstrated to be a reasonable tool for both confirmation and exclusion of present diagnoses. This reinforces the applicability of the prototype-based definition of diagnoses in clinical practice. Further confirmation of these findings would help to bring research to a more clinical practice-based scenario, reducing the distance between research and practice.

## Data Availability Statement

The raw data supporting the conclusions of this article will be made available by the authors, without undue reservation.

## Ethics Statement

The studies involving human participants were reviewed and approved by Comitê de Ética e Pesquisa do Instituto de Psiquiatria da UFRJ. The patients/participants provided their written informed consent to participate in this study.

## Author Contributions

HR elaborated the methodology, trained the residents for data collection, organized the data, conducted the statistical tests, and wrote the text. TS, LK, AP, BS, and CV collected the data, organized the data sheets, and reviewed the subjects' diagnoses. DT-C and MC oriented the methodology development and resident training, reviewed the draft, and edited the final text. All authors contributed to the article and approved the submitted version.

## Funding

HR receives a monthly wage from a PhD Grant offered by CAPES, Education Ministry, Brazil. TS receives a monthly wage from a Scientific Initiation Program Grant offered by CAPES, Education Ministry, Brazil.

## Conflict of Interest

The authors declare that the research was conducted in the absence of any commercial or financial relationships that could be construed as a potential conflict of interest.

## Publisher's Note

All claims expressed in this article are solely those of the authors and do not necessarily represent those of their affiliated organizations, or those of the publisher, the editors and the reviewers. Any product that may be evaluated in this article, or claim that may be made by its manufacturer, is not guaranteed or endorsed by the publisher.
